# Charitable Dispensaries of Bengal

**Published:** 1899-04-15

**Authors:** 


### CHARITABLE DISPENSARIES OF BENGAL.

The most pleasing feature of the latest report upon the Charitable Dispensaries of Bengal is the unusually large addition which has been made in the year 1897 to those institutions which are maintained from local funds. Fifteen dispensaries of this class have been opened during the twelve months, showing the increasing interest local bodies are taking in bringing medical relief within easy reach of the people. The District Board of Backergunge, in particular, have been most active in this work, having established eleven dispensaries in different parts of the district in two years, and in other places where they have not yet been able to set up dispensaries they supplied medicine chests containing simple remedies to the village headman for the treatment of the sick in the localities. In this way much medical relief has been given. There is also abundant evidence of the growing popularity of these dispensaries throughout the whole of the province of Bengal. The increase amongst male patients in 1897 shows a total of 108,209, and the number of females was greater by 35,432, and children by 5,861. In 1891 a plan for cottage wards to give privacy to female patients was ordered to be adopted in Bengal, and recently a new plan for dispensaries, prepared after the model in the central provinces, was circulated to civil surgeons for adoption when fresh buildings are constructed. This plan provides for the separate treatment of female patients both in the indoor and outdoor departments, which is most desirable, and should lead, as the authorities expect, to a large increase in the number of females requiring relief, though the number who sought the dispensaries during 1897 was 35,432 in excess of 1896. The greatest difficulty which the Inspector-General has to contend with is the regular visitation of the Bengal dispensaries. Every dispensary should be inspected four times a year by the Civil Surgeon, but in many instances last year this was not done. The fault does not lie entirely with the surgeons themselves, although the Inspector-General maintains that if the tours were arranged in a more systematic manner it would be possible to accomplish much more than is done at present without detriment to other duties. But when one reads of a dispensary "50 miles from head-quarters, the road being impassable during the greater part of the year, and no rest house available," it is easy to understand that one visitation was all that was accomplished. Another dispensary is "18 miles from the Hilly Railway Station, and no conveyance is to be had." Yet a third is "too far for a boat journey, except in the rains, and by road too difficult of approach except in very dry weather. Twice an attempt was made, but each time an accident prevented." The earthquake destroyed the roads in some places so completely that it was quite impossible to traverse them at all. All these difficulties to surmount may make the life of a civil surgeon in India less humdrum than that of a country practitioner in England, but assuredly they render the work considerably more arduous.

